# The Risk-based Treatment of Acute Pulmonary Embolism

**DOI:** 10.4021/jocmr2009.03.1229

**Published:** 2009-03-31

**Authors:** Luca Masotti, Annalisa Mannucci, Fabio Antonelli, Vincenzo Maurini, Roberto Testa, Sergio Marchetti, Giancarlo Landini, Roberto Cappelli

**Affiliations:** a Internal Medicine, Cecina Hospital, Italy; b Clinical Chemistry, Cecina Hospital, Italy; c Emergency Room, Cecina Hospital, Italy; d Cardiology, Cecina Hospital, Italy; e Radiodiagnostics, cecina Hospital, Italy; f Internal Medicine, Santa Maria Nuova Hospital, Florence, Italy; g Thrombosis Center, University of Siena, Siena, Italy

## Abstract

Risk evaluation and prognostic stratification based upon clinical and radiological findings and new cardiac biomarkers, such as natriuretic peptides (NP) and troponins, represent key points in modern management of acute pulmonary embolism (PE). Literature evidence shows that normotensive PE with right heart dysfunction (RHD), defined as submassive PE, has poorer prognosis when compared to normotensive PE without RHD, defined as non-massive PE; thus whether submassive PE should be managed more aggressively and with closer monitoring represents the crucial question about acute PE treatment. Although the answer is yet unclear, the most recent guidelines address to thrombolysis as treatment choice in selected high risk patients with submassive PE. Guidelines also clarify the indications for unfractioned and low molecular weight heparins and fondaparinux. Therefore, in the present article, the authors focus on modern risk-based therapeutic guidelines of acute PE.

**Keywords:**

Pulmonary embolism; Treatment; Prognosis; Biomarkers; Chocardiography; Hemodynamic; Guidelines

## Introduction

Pulmonary embolism (PE) is a frequent disorder in clinical practice with a hospital incidence of 0.4% of all admissions [1]. Mortality rate varies according to clinical presentation, in fact, it is about 2% in patients who appear normotensive without right heart compromise, it increases to 10-15% in normotensive patients with right heart compromise, and reaches around 30% in patients who present with cardiogenic shock and 70% in patients who present with cardiac arrest [2]. Treatment is usually effective when properly and quickly started; it should be kept in mind that about 10% of patients died within the first hour from onset of symptoms [2]. Thus early diagnosis, quick and appropriate treatment customized according to strict clinical and prognostic evaluation of the patient, are crucial moments in the modern therapeutic approach to this disease.

Since many years, thrombolysis was recognized to find indications in haemodinamically unstable patients with PE, in other words, in patients with shock (systolic blood pressure, SBP, under 90 mm Hg). Up to now, the big question on therapy has been represented whether or not thrombolysis was also indicated in normotensive patients with evidence of right heart dysfunction (RHD) who have a significantly worse prognosis than normotensive patients without RHD. In the MAPPET III Study (Management Strategies And Prognosis of Pulmonary Embolism Trial-3), Konstantinides et al. had shown that, although in normotensive patients with RHD (condition defined as submassive-intermediate risk PE, see later), treatment with thrombolysis reduced mortality in a non-significant percentage, 3.4% in patients treated with unfractionated heparin (UFH) vs 2.2% in patients thrombolysed, p = ns, compared with more bleeding events in patients treated with thrombolysis; at the same time; thrombolytic treatment, added in patients initially treated with UFH with subsequent evolution toward hemodynamic instability (rescue treatment), clearly and significantly reduced mortality compared to patients not undergoing this treatment in the event of deteriorating hemodynamics (mortality 10.2 vs. 24.6% respectively, p<0.05) [3]. Therefore, this trial had established the role of thrombolysis as rescue therapy in normotensive patients with RHD and initially treated with UFH that evolves towards hemodynamic instability, but thrombolysis was not recommended by the VII Edition guidelines of American College of Chest Physicians (ACCP) in normotensive patients with RHD [4]. The increase of scientific evidence in favor of increased risk of mortality in patients with RHD without shock or hypotension at presentation and especially the considerable amount of evidence relating to the role of some prognostic biomarkers recently introduced (troponins and natriuretic peptides) complementary to the diagnostic imaging (echocardiography and computer tomography pulmonary angiography, CTPA) resulted in the development of new therapeutic guidelines of PE in the acute phase, based on the estimation of clinical risk and prognostic stratification much recently published (European Society of Cardiology, ESC, August 2008, ACCP, VIII Edition, June 2008), which will be discussed in this article [5, 6].

## Haemodinamic consequences of PE

**Figure 1:**
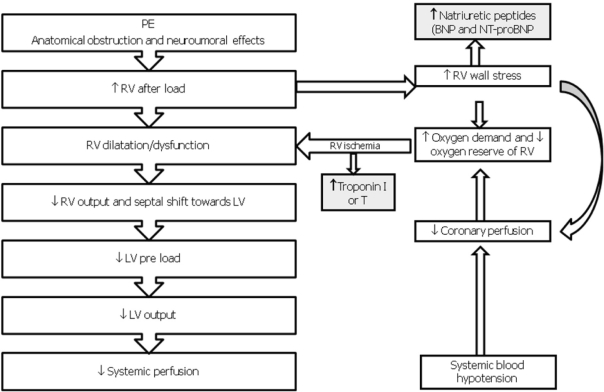
Pathophysiology of hemodynamic instability due to PE. RV, Right Ventricle; LV, Left Ventricle; BNP, Brain Natriuretic Peptide; NT-proBNP, AminoTerminal-proBrain Natriuretic Peptide.

The extension of obstructed pulmonary arterial bed, the pre-existence of cardio-pulmonary disease and response to the release of vasoactive substances determined by local thrombus influence the pathophysiological response in acute PE [2, 7]. The obstruction, caused by mechanical thrombus associated with pulmonary vasoconstriction determined by the release of vasoactive substances and hypoxia, leads to an increase of pulmonary vascular resistances and after-load of the right ventricle. This increase may result in right heart dilation, myocardial ischemia and hypokinesia, trcuspidal regurgitation or insufficiency, and finally right ventricular failure. In some patients, a rapid deterioration in progressive systemic arterial hypotension, cardiogenic shock and cardiac arrest may occur. Approximately 5-10% of patients with normal blood pressure at presentation have a rapid deterioration in the early stages of the hospital admission, and this event is due to the recurrence of embolisation or to the acute dysfunction of the right ventricle [2, 7]. The hemodynamic consequences of PE are summarized in Figure 1. The respiratory consequences of PE are represented mainly by mismatch ventilation/perfusion (pulmonary areas ventilated but not perfused), increased of total dead space and right-left shunts [2, 7].

## Old and new clinical classification of PE

PE is classically divided into massive form when manifested by cardiac arrest, cardiogenic shock or arterial hypotension (haemodynamically unstable form) form, sub-massive (PE with RHD but without hemodynamic instability) and non-massive form (PE without RHD and without hemodynamic instability, usually caused by an obstruction of pulmonary arterial bed < 30%) [2, 6, 8, 9].

Most recently, European Society of Cardiology (ESC) proposed to divide the clinical pictures of PE on the basis of clinical risk and prognosis; it follows that PE is divided into high risk (previous massive PE, mortality > 15% in the acute phase) and not high risk forms [5]. The latter is divided into intermediate risk form (previous sub-massive PE, mortality 3-15%) and low-risk form (previous non-massive PE, mortality < 3%). Table 1 summarizes the ESC classification of PE.

**Table 1:** Clinical classification of PE

ATS 1999, ESC 2000, BTS 2003, ACEP 2003, ACCP 2004, ACCP 2008 MASSIVE (cardiac arrest, shock, hypotension) SUB-MASSIVE (normotensive PE with RHD ) NON MASSIVE (normotensive PE without RHD)
ESC 2008 HIGH RISK (cardiac arrest, shock, hypotension) NON HIGH RISK INTERMEDIATE RISK (normotensive PE with RHD and/or high BNP and/or high troponins) LOW RISK (normotensive PE without RHD and low BNP and low troponins)

ATS, American Thoracic Society; ESC, European Society of Cardiology; BTS, British Thoracic Society; ACEP; American College of Emergency Physicians; ACCP; American College of Chest Physicians; RHD; right heart dysfunction.

## Risk assessment and prognostic stratification

Patients over 75 years, bed rest over four days, cancer, chronic obstructive pulmonary disease, heart failure, kidney failure, tachycardia and syncope are all clinical and comorbidity indicators of poor prognosis in acute PE [10, 11].

However the most important negative prognostic indicator in patients with PE is shock at presentation or during the acute phase of illness [2]. The ESC defines high risk PE as when it occurs with shock or arterial hypotension (SBP < 90 mm Hg); the relief of RHD and elevated biomarkers is not necessary to define the high risk [5]. It was recently introduced a parameter defined as shock index (ratio between heart rate in beats per minute and SBP in mm Hg), it is easy to apply in clinical practice. A value of shock index > 1 correlates with the echocardiographic presence of RHD and increased pulmonary arterial systolic pressure (sPAP) and it is associated with increased hospital and thirty-days mortality [12, 13].

Pulmonary Embolism Severity Index (PESI) is a score that identifies 5 classes with a growing risk of mortality according to a strictly clinical parameters [14]. The patients in class I - II (low risk, mortality ≤ 1.2%) may receive less aggressive therapy (subcutaneous low molecular weight heparins, LMWH, or fondaparinux and vitamin K antagonists) and they could be discharged from hospital more quickly or even could receive home treatment, whereas patients in class III - IV (mortality rate 4.8, 13.6, 25%, respectively) should receive more aggressive treatment and close monitoring [14].

The electrocardiographic findings more indicative of negative prognosis in acute PE are the presence and number of negative T waves on precordial leads [15]. The traditional parameters offered by arterial blood gas analysis do not seem to offer advantages in terms of prognosis.

Trans-thoracic echocardiography is the golden diagnostic standard for identifying RHD [16, 17]. The main echocardiographic findings of RHD are represented by hypokinesia of right ventricle (mild, moderate, severe), dilatation of right ventricle (four chambers end-diastolic right ventricle/left ventricle diameter ratio > 1), and findings of pulmonary arterial hypertension [17]. The presence of RHD at echocardiography is related to a negative prognosis both in shock patients and in normotensive patients [11, 17].

About 80% of patients with PE are normotensive at presentation; from one third to half of normotensive patients have echocardiographic RHD [10, 18]. In normotensive patients without RHD the range of hospital mortality is 0 - 9.6%, while in patients with RHD is 11.8 - 23% [19]. Normotensive patients with echocardiographic RHD have also reduced survival of 30 days (mortality increased to 17%) compared to patients without RHD [18]. The presence of RHD at presentation correlates also with poor pulmonary thrombi dissolution at six months and with the greatest incidence of recurrence of venous thromboembolic events [20].

Computer tomography pulmonary angiography (CTPA) has become the golden standard diagnostic method for PE. It may give also prognostic information. By using information derived from CTPA imaging it is possible to consider 3 indices of PE severity: the CTPE index (Computer Tomography Pulmonary Embolism index), which assesses the degree of obstruction and the number of pulmonary arteries affected by PE [21]; the right ventricle/left ventricle diameter ratio assessed by CTPA, when increased more than 1, it seems to correlate with a negative prognosis [22]; and a new index much recently proposed by Ghanima et al, which assesses the degree of proximality of pulmonary arteries involvement in respect to main pulmonary artery [23]. All these indexes correlate with severity and prognosis of PE, but in literature there are no consistent data for their use as a prognostic tool in routine clinical practice.

Among the biomarkers used for prognostic stratification of PE, with proven effectiveness are the results of troponins assay (I and T) and natriuretic peptides (brain natriuretic peptide or its terminal portion, BNP and NT-proBNP respectively). The increase of troponins indicates myocardial damage and the expression of microinfarcts of right ventricular wall, while the natriuretic peptides are indices of RHD and their increase is attributable to stress of right ventricular wall [5]. The increase in these biomarkers was related to echocardiographic and CTPA data of RHD, it has negative predictive value [24]. Recent meta-analysis confirms that the increase in troponin I and T and natriuretic peptides is predictive of adverse prognosis in terms of mortality and morbidity in the acute phase of PE [25-27]. Hence, the rational basis for the introduction of these biomarkers is complementary to echocardiographic data in the evaluation of the clinical severity and prognostic stratification of PE made by ESC [5]. Table 2 summarizes the classes of risks suggested by ESC together with the parameters used to define them. It is possible to notice that the intermediate class at risk, for which therapy is most debated, provides for the possibility of three combinations arising from the presence or absence of RHD at echocardiography, increased natriuretic peptides and/or troponins.

**Table 2:** ESC criteria for identifying the prognostic risk of PE

Risk (mortality in acute phase %)	Shock/hypotension	Echocardiographic and biomarkers findings of RHD (↑BNP or NTpro-BNP)	Findings of myocardial injury: ↑ troponin I or T
High (15%)	Present	Present*	Present*
Intermediate (3 - 15%)	Absent Absent Absent	Present Absent Present	present Present Absent
Low (< 3%)	Absent	Absent	Absent

*generally present but not necessary to define high risk

Finally other biomarkers evaluated in terms of prognosis are D-dimer, its values seem to be correlated linearly with the commitment thrombotic proximal pulmonary artery and the clinical severity of PE [28], which, however, being very sensitive and relatively little specific, is unlikely as considerable prognostic index. The hearty type fatty acid binding proteins (htFABP) whose growth seems to correlate better than BNP and troponins with the prognosis of PE in the acute phase [29], but at the moment they are not widely disseminated in the hospital laboratories.

## Modern guidelines for antithrombotic therapy in the acute phase of PE

**Figure 2:**
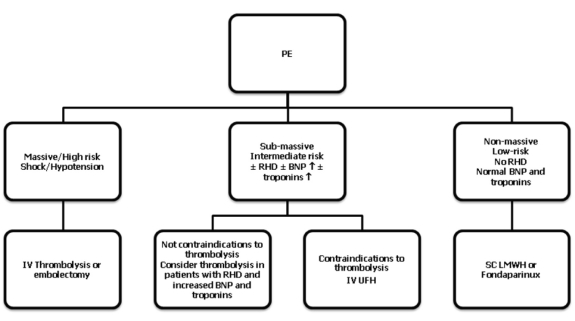
Summary of PE treatment according to modern guidelines. RHD, right heart dysfunction; BNP, brain natriuretic peptide; IV, intravenous; SC, subcutaneous.

Modern treatment guidelines proposed by ESC and ACCP indicate that the treatment of acute PE is based on the clinical risk and prognostic stratification, it is based on pharmacological and non-pharmacological strategies. Figure 2 summarizes the antithrombotic treatment of PE depending on the category of risk.

First of all, patients with high probability of clinical PE should receive an intravenous bolus of UFH, less than contraindications, when they undergo inspection for confirmation.

In patients with massive/high-risk PE and hemodynamically unstable, thrombolytic therapy has shown to improve more quickly hemodynamic compromise, while it is uncertain about long-term outcomes [5, 6]. Among the possible thrombolytic agents, streptokinasis, urokinasis and recombinant tissue plasminogen activator (rtPA, alteplase), the last one is preferable. Schemes of thrombolytic therapy with infusions of short duration (≤ 2 h) are suggested compared to longer infusions (12 - 24 h) that do not have beneficial effects on hemodynamics and that are burdened by higher bleeding complications. The rtPA is administered by bolus of 10 mg IV, followed by 2 hours in a dose determined by body weight; it must not be exceeded the total dose of 100 mg. In cases of extreme severity, it can be administered by dose of 0.6 mg/kg of bolus rtPA IV in rapid 5 - 15 minutes, but not exceeding the maximum dose of 50 mg IV. It should be noted that during the infusion of rtPA, The UFH infusion should be discontinued and then resumed after the infusion of thrombolytic [5, 6].

Thrombolytic treatment is indicated (recommendation IIB) in selected patients with sub-massive PE/intermediate risk who have evidence of RHD echocardiographically and by biomarkers (BNP or NTproBNP), myocardial damage shown by increased levels of troponin I or T [5, 6] and not absolute contraindications to thrombolysis (Table 3).

**Table 3:** Contraindications to thrombolysis in PE

Absolute Haemorragic stroke or stroke of unknown origin at any time Ischemic stroke within six months Central nervous system damage or cancer Recent major trauma/surgery/head injury within three weeks Gastrointestinal bleeding within last months Known bleeding
Relative Age over 85 years Transient ischemic attacks in the previous six months Vitamin K antagonists treatment Traumatic cardio-pulmonary resuscitation Non compressible punctures within 30 days Refractary hypertension (systolic blood pressure > 180 mm Hg) Pregnancy or within one week post-partum Infective endocarditis Advanced liver disease Active peptic ulcer

Intravenous UFH should be reserved in patients with sub-massive PE/intermediate risk where there are contraindications to thrombolysis. A bolus of IV UFH (in the vast majority of cases are made 5000 IU IV bolus, in elderly patients might be prudent a bolus of 3000 IU) followed by continuous infusion of UFH with infusion pump or syringe pump in a dose would lead to an increase of aPTT (time of activated partial thromboplastin) of 1.5 - 2.5 times the normal value, monitored by the aPTT nomograms [5, 6].

In patients with non-massive/low-risk PE, the subcutaneous LMWH or fondaparinux at anticoagulant dose are recommended (example enoxaparin 100 IU/kg twice daily, 5 mg fondaparinux if weight < 50 Kg, 7.5 mg if weight between 50 and 100 kg, 10 mg if weight > 100 kg in single dose). LMWH should be avoided in patients with renal failure and/or severe obesity; in the first situation the choice is UFH, in obese patients the choice is subcutaneous fondaparinux at dosage of 10 mg/day. LMWH should be continued for the first 3 - 6 months for prevention of secondary venous thromboembolism in patients with cancer. Fondaparinux is further indication in patients with heparin induced thrombocytopenia. The intravenous UFH, LMWH and fondaparinux should be administered at least for five consecutive days, unless complications occurring, overlapping vitamin K antagonists (VKA) [5, 6]. VKA should be started in the same day of heparins or fondapariunx, overlapping it, with the aim of achieving a therapeutic range to maintain the value of INR (International Normalized Ratio) in the range 2 - 3 (target 2.5) in most patients. Heparins or fondaparinux should be discontinued when the value of INR is included in the therapeutic range for at least two consecutive days [5, 6].

The indication for the positioning of vena cava filters are limited to situations where VKA are absolutely contraindicated or VKA should be discontinued for the appearance of complications, or if PE has occurred in patients under VKA well conducted (INR in the therapeutic range). Vena cava filter could find indication in patients undergoing pulmonary embolectomy or thromboendoarterectomy for chronic thromboembolic pulmonary hypertension [5, 6].

Surgical or mechanical embolectomy is indicated in patients with massive/high risk PE, where there are absolute contraindications to thrombolysis, or this was ineffective after documentation of pulmonary thrombus through trans-esophageal echocardiogram or pulmonary arteriography. Pulmonary thromboendoarterectomy is indicated in chronic tromboembolic pulmonary hypertension [5, 6].

## Conclusions

PE is a common disease in clinical practice, burdened by high morbidity and mortality especially if combined with hemodynamic instability. Modern guidelines based on the risk estimate according to clinical and instrumental indicators and biomarkers can customize treatment with a good chance of success, with minimum complications. The most important news on treatment is the possibility of thrombolysis in selected normotensive patients with echocardiographic and laboratory findings which are at high risk of adverse prognosis. This strategy will be evaluated in prospective clinical multicenter intervention trials which will determine their effectiveness. The current trial undergoing is an important European trial (PEITHO Study) whose results are expected in the coming years and that will give important answers to the previous questions [30].
